# A multilectin affinity approach for comparative glycoprotein profiling of rheumatoid arthritis and spondyloarthropathy

**DOI:** 10.1186/1559-0275-10-11

**Published:** 2013-09-06

**Authors:** Mitali Bhattacharjee, Rakesh Sharma, Renu Goel, Lavanya Balakrishnan, Santosh Renuse, Jayshree Advani, Shantal Tankala Gupta, Renu Verma, Sneha M Pinto, Nirujogi Raja Sekhar, Bipin Nair, T S Keshava Prasad, H C Harsha, Ramesh Jois, Subramanian Shankar, Akhilesh Pandey

**Affiliations:** 1 Institute of Bioinformatics, International Technology Park, Bangalore 560066, India; 2 Amrita School of Biotechnology, Amrita Vishwa Vidyapeetham, Kollam 690525, India; 3 Department of Neurochemistry, National Institute of Mental Health and Neuro Sciences, Bangalore 560029, India; 4 Department of Biotechnology, Kuvempu University, Shankaraghatta 577451, India; 5 Department of Internal Medicine, Armed Forces Medical College, Pune 411040, India; 6 Manipal University, Madhav Nagar, Manipal 576104, India; 7 Centre of Excellence in Bioinformatics, Bioinformatics Centre, School of Life Sciences, Pondicherry University, Puducherry 605 014, India; 8 Department of Rheumatology, Fortis Hospital, Bangalore 560076, India; 9 McKusick-Nathans Institute of Genetic Medicine, Johns Hopkins University School of Medicine, 733 N. Broadway, Baltimore, MD 21205, USA; 10 Department of Biological Chemistry, Johns Hopkins University School of Medicine, 733 N. Broadway, Baltimore, MD 21205, USA; 11 Department of Pathology, Johns Hopkins University School of Medicine, 733 N. Broadway, Baltimore, MD 21205, USA; 12 Department of Oncology, Johns Hopkins University School of Medicine, Baltimore, Maryland 21205, USA

**Keywords:** Pannus, Prognostic marker, Endothelial dysfunction, Synovium, Biomarkers

## Abstract

**Background:**

Arthritis refers to inflammation of joints and includes common disorders such as rheumatoid arthritis (RA) and spondyloarthropathies (SpAs). These diseases differ mainly in terms of their clinical manifestations and the underlying pathogenesis. Glycoproteins in synovial fluid might reflect the disease activity status in the joints affected by arthritis; yet they have not been systematically studied previously. Although markers have been described for assisting in the diagnosis of RA, there are currently no known biomarkers for SpA.

**Materials and methods:**

We sought to determine the relative abundance of glycoproteins in RA and SpA by lectin affinity chromatography coupled to iTRAQ labeling and LC-MS/MS analysis. We also used ELISA to validate the overexpression of VCAM-1, one of the candidate proteins identified in this study, in synovial fluid from RA patients.

**Results and discussion:**

We identified proteins that were previously reported to be overexpressed in RA including metalloproteinase inhibitor 1 (TIMP1), myeloperoxidase (MPO) and several S100 proteins. In addition, we discovered several novel candidates that were overexpressed in SpA including Apolipoproteins C-II and C-III and the SUN domain-containing protein 3 (SUN3). Novel molecules found overexpressed in RA included extracellular matrix protein 1 (ECM1) and lumican (LUM). We validated one of the candidate biomarkers, vascular cell adhesion molecule 1 (VCAM1), in 20 RA and SpA samples using ELISA and confirmed its overexpression in RA (p-value <0.01). Our quantitative glycoproteomic approach to study arthritic disorders should open up new avenues for additional proteomics-based discovery studies in rheumatological disorders.

## Background

Bone is a specialized form of connective tissue which undergoes continuous remodelling throughout an individual’s life span [1]. This involves osteoclast-based removal of mineralized bone which is balanced by osteoblast-based bone mineralization [1]. The entire process of bone remodeling is regulated by several factors including immune mediators [1,2]. In rheumatologic disorders, aberrant presence of these regulators may either lead to progressive and irreversible bone erosion or abnormal bone formation [1,2]. Rheumatoid arthritis (RA) and spondyloarthropathies (SpA) are two chronic multi-system and complex autoimmune inflammatory disorders which are considerably affected by an abnormal bone remodelling cycle [2,3]. RA is characterized by excessive bone degradation with relatively low bone formation targeting the small joints of the body in a symmetrical pattern [2,4]. SpA, on the other hand, encompasses a number of disease subtypes including ankylosing spondylitis, reactive arthritis, arthritis associated with inflammatory bowel disease, psoriatic arthropathy and undifferentiated spondyloarthropathy [5]. Essentially, the major pathological changes in SpA are characterized by an aberrant bone formation that predominantly affects the spine and large joints asymmetrically [6,7]. The diseases are associated with high morbidity due to pain and of restriction of joint movements resulting in depreciation of quality of life. In addition, these inflammatory autoimmune disorders are associated with increased mortality and reduced life span of almost 10–12 years resulting from cardiovascular and renal complications [8-11]. In light of the significant morbidity and mortality of rheumatological disorders, research into discovering biomarkers for early detection, differential diagnosis, prognosis and response to therapy is critical [12]. Despite the availability of multiple markers for the diagnosis of RA, their performance leaves room for discovering additional biomarkers with better sensitivity and specificity [13]. There are no molecular markers available for the diagnosis of SpA although expression of HLA-B27 has been shown to be associated with development of SpA [14]. Thus, the diagnosis of both of these disorders is largely made based on clinical criteria with serological and radiological markers providing supportive evidence [14,15].

Generally, disease marker proteins secreted into the bloodstream by affected tissues or cells are expected to be present in relatively low concentration [16-18]. In contrast, proximal fluid obtained from the affected tissue/organ serve as the local environment where the disease manifests and are preferable for discovering disease marker proteins as they are likely to be more abundant [16-18]. In the field of rheumatology, the ideal proximal fluid is the synovial fluid collected by aspiration of affected joints [12]. The hyaluronic acid rich fluid produced by synovial membrane is an ultrafiltrate of blood released from the dense networks of capillaries present in the synovium [19,20]. This fluid is a lubricant and provides nutrients to cells and tissues of the joints [21]. In the site of pathogenesis, mostly the knee joint, fluid accumulation increases with the severity of the disease [21].

Identification and validation of protein markers in synovial fluid using mass spectrometry is challenging and the major constraint is perhaps the dynamic and complex nature of this fluid, which increases with inflammation of the synovium [19,22-24]. Previous reports have indicated an increased permeability of synovial membrane for selected plasma proteins during disease states, the majority of which are glycoproteins [25]. Glycoproteins are vital in many biological processes and have been considered critical for biomarker discovery to monitor disease progression and treatment [26]. Disease activity status of a patient could be monitored through the detection of specific glycoproteins released from affected tissues or cells into the proximal fluid [27]. Glycoproteins in particular, have been found to be overexpressed in serum and synovial fluid of RA patients compared to healthy individuals and have been considered critical for rheumatic diseases [28,29]. Considering the functional importance and applications in biomarker discovery, we sought to determine the relative abundance of glycoproteins across RA and SpA. Essentially, differential expression patterns of proteins can be determined with iTRAQ, ICAT, SILAC or ^18^O labeling methods, among others [30-33]. To this end, we carried out multilectin affinity-based glycoprotein enrichment from synovial fluid followed by studying protein abundance patterns across RA and SpA by using an iTRAQ-based quantitative proteomics strategy. To our knowledge, this is the first quantitative glycoprotein profiling study of synovial fluid samples. A similar approach of comparative glycoprotein profiling by ^18^O labeling in hepatocellular cancer tissues and plasma samples by our group has already been reported [32].

In this study, we observed several previously reported marker proteins in addition to a number of novel proteins which could potentially accelerate biomarker discovery in rheumatologic diseases. Additionally, using ELISA, we validated the overexpression of the glycoprotein, VCAM-1, in RA. The use of comparative glycoproteomics for discovering biomarkers and therapeutic targets represents a novel approach that could be generally applied to a spectrum of autoimmune disorders.

## Results and discussion

**Figure 1:**
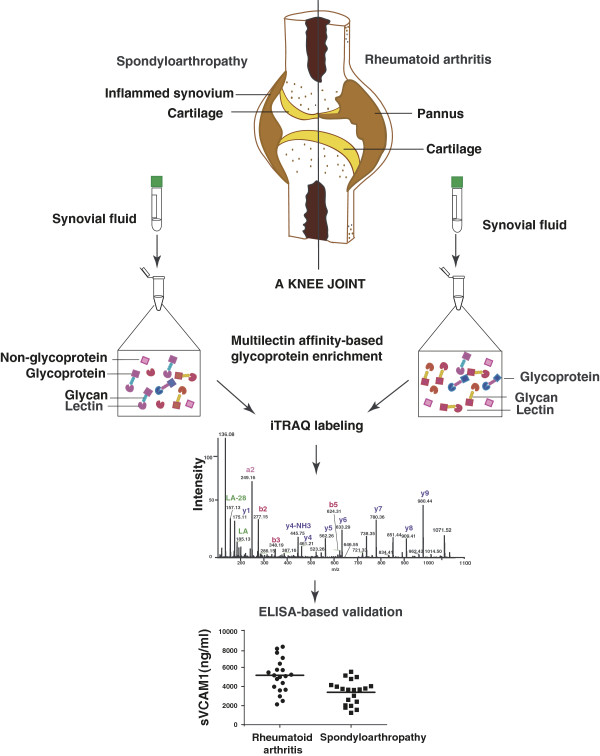
**Outline of the strategy implemented in the study.** Synovial fluid samples from RA and SpA patients were subjected to multilectin affinity enrichment. Three lectins - Concanavlin A (C), Wheat Germ Agglutinin (W) and Jacalin (J) were pooled together and used for glycoprotein enrichment from synovial fluid samples of RA and SpA. The enriched proteins were trypsin digested followed by iTRAQ labeling (RA with 116 and SpA with 117). The labeled tryptic peptides were pooled and fractionated by SCX chromatography. The samples were then analyzed on an LTQ-Orbitrap Velos mass spectrometer coupled to a nano-HPLC unit. Data obtained were searched using SEQUEST and Mascot. Finally, an ELISA assay was carried out to validate the upregulation of sVCAM-1 in RA as compared to SpA.

**Figure 2:**
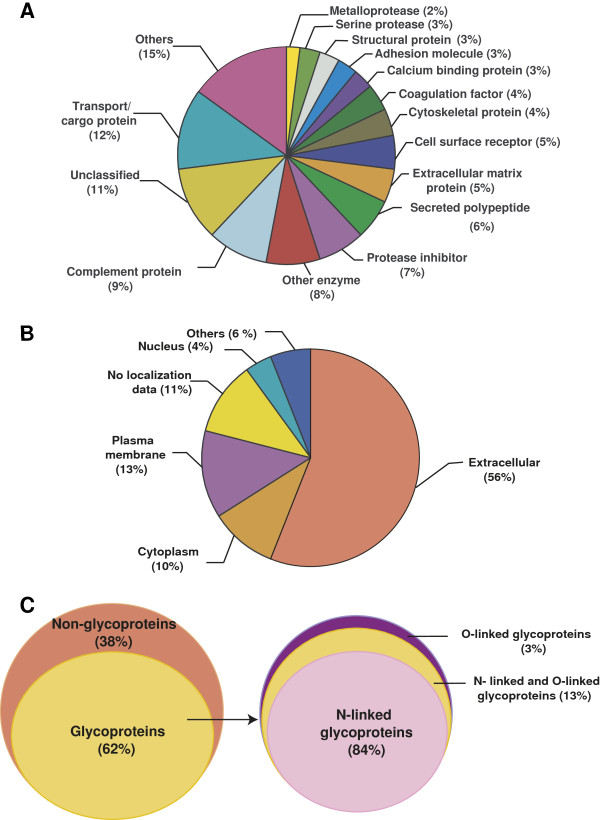
**Summary of bioinformatics analysis.** Protein distribution based on molecular class **(A)** and subcellular localization **(B)**. Secretory and membrane proteins were observed in the study and the majority was extracellular. Distribution of glycoproteins and non-glycosylated proteins, as illustrated in the form of pie charts **(C)** with an inset representing the overall distribution of the N-linked glycoproteins, O-linked and those with both types of linkages. From the total number of proteins identified, 62% are glycoproteins and 38% are non-glycoproteins.

This study was conducted to identify differentially regulated glycoproteins between the two chronic diseases, RA and SpA. Because aspiration of synovial fluid samples from healthy individuals is not permitted for ethical reasons [34] and because molecular markers are required to distinguish different types of arthritis from each other and not from unaffected cases, we chose to compare RA with SpA. The strategy employed in this study is shown in Figure 1. Through this approach, we identified a total of 210 proteins out of which 164 proteins were quantified (35 proteins were identified from single peptide hits with more than one peptide*-*spectrum match (PSM) while the rest were with two or more peptide hits). From the list, 70 showed a ≥1.5-fold difference between the two groups (combined protein and peptide lists are provided in Additional file 1: Table S1). Gene Ontology-based molecular class and subcellular localization of the identified proteins are shown in Figures 2(A) and (B), respectively, and details provided in Additional file 2: Table S2.

### Enrichment of glycoproteins

A combination of multiple lectins with different glycan specificities improves the repertoire of proteins containing both N- and O- linked glycoproteins [32]. Thus, we combined three types of lectins, Concanavalin A, Wheat germ agglutinin and Jacalin, to enhance the coverage of glycoproteins captured from the synovial fluid of RA and SpA patients. Of the 210 proteins identified, 131 (62%) were already reported to be glycosylated. The distribution is as illustrated in Figure 2 (C) and Additional file 2: Table S2.

### Differentially expressed proteins identified in this study

The number of upregulated proteins in RA as compared to SpA was 44 while 26 proteins were more highly expressed in SpA. Partial lists of these upregulated proteins are provided in Tables 1 and 2, respectively. A brief description of the biological role of the different classes of proteins identified in our study is provided below.

**Table 1:** A partial list of proteins upregulated in RA as compared to SpA

**Gene symbol**	**Protein**	**Fold-change (RA/SpA)**	**Molecular class**	**Number of unique peptides**	**Whether already known glycoprotein**
*MPO*	Myeloperoxidase	3.15	Oxidoreductase	2	Yes; N-linked
*LUM*	Lumican	2.13	Extracellular matrix protein	8	Yes; N-linked
*TIMP1*	Metalloproteinase inhibitor 1	1.74	Extracellular matrix protein	1	Yes; N-linked
*S100A9*	Protein S100-A9	4.73	Calcium ion binding protein	4	No

**Table 2:** A partial list of proteins upregulated in SpA as compared to RA

**Gene symbol**	**Protein**	**Fold-change (SpA/RA)**	**Molecular class**	**Number of unique peptides**	**Whether already known glycoprotein**
*APCS*	Serum amyloid P-component	3.44	Secreted polypeptide	4	Yes; N-linked
*C5*	Complement C5	4.16	Complement protein	10	Yes; N-linked
*LGALS3BP*	Galectin-3-binding protein	4.16	Extracellular matrix protein	2	Yes; N-linked
*SAA1*	Serum amyloid A protein	2.77	Transport/cargo protein	2	No

### Extracellular matrix proteins

Lumican (LUM) is a major proteoglycan component that binds to collagens in bones and its secretion reflects bone repair [35]. It has been implicated as an atherosclerotic marker that induces collagen fibrillogenesis in coronary atherosclerosis [36]. It was found to be 2-fold upregulated in RA (see Additional file 3: Figure S1A for a representative MS/MS spectrum) and has not previously been linked to this disease. Extracellular matrix protein1 (ECM1), which was >1.5-fold upregulated in RA may be considered as a novel matrix marker protein in RA. It is involved in regulation of endochondral bone formation and activation of the endothelial cell proliferation, thus inducing angiogenesis [37]. This family of extracellular matrix proteins is already reported to be widely studied in atherosclerosis [36,38]. Further, upregulation of Keratin Type I cytoskeletal protein 14 (KRT14) by 2-fold in RA is in keeping with the already known overexpression of cytokeratins in synovial membranes of RA patients [39].

### S100 protein family

We identified both S100A8 and S100A9 proteins as > 4-fold upregulated in RA. They are acidic proteins released by neutrophils and macrophages [40]. In RA, formation of the S100A8/S100A9 complex, also referred to as calprotectin, has been observed to significantly increase with the severity of the disease [41,42]. It has been implicated in the conversion of normal synovium to a pseudotumoral one called pannus through activation of the Receptor for Advanced Glycation End products (RAGE) protein localized on synoviocytes, which is a receptor for S100 proteins [42]. An MS/MS spectrum of one of the representative peptides of protein S100A8 is shown in Additional file 3: Figure S1(B).

### Inflammatory mediators

Among the complement proteins, the notable component identified was complement protein C3. This protein was identified to be 5-fold upregulated in SpA. C3 protein from synovial fluid of SpAs has been already observed to be higher than in RA [43]. It is also known to be elevated in ankylosing spondylitis and decreased in RA compared to unaffected joints [44,45]. C3 is a central protein involved in the classical pathway of complement activation [46]. Complement component proteins have been considered as potential diagnostic markers for SpAs, which is in keeping with our findings [43]. CD44, commonly known as hyaluronic acid receptor, is an inflammatory marker that has been studied in RA was found to be 2-fold upregulated in RA [47]. It is also referred as osteocyte differentiation marker and plays a significant role in the inhibition of osteoclast differentiation [48]. Myeloperoxidase (MPO) was found to be 3-fold upregulated in RA and has been implicated in tissue damage caused by the release of oxidative radicals usually from neutrophils in RA patients [49].

Complement C5, the fifth component of the complement family of proteins, was observed to be upregulated by 4-fold in SpA (see a representative MS/MS spectrum in Additional file 3: Figure S1(C). In association with other complement proteins, C6, C7 and C8, its proteolytic fragment C5b forms a membrane attack complex to carry out cell lysis of pathogens [50]. It has not been previously associated with SpAs.

### Vascular adhesion molecules

Vascular cell adhesion molecule 1 (VCAM1), was found to be 2-fold overexpressed in RA. Its expression, both at the mRNA level in synovial tissues and at the protein level in various sites including synovial membrane, synovial fluid and serum of RA cases, has already been reported [51-53]. In addition to its role in inflammation, VCAM-1 has also been implicated in angiogenesis [54] and in atherosclerosis [55]. We also identified the atherosclerotic marker, vascular endothelial-cadherin designated as cadherin 5 (CDH5), to be >1.5-fold upregulated in RA, providing further evidence for the association of atherosclerosis with RA [56]. In a previous study, TNF α stimulation was reported to induce the release of secretory form of this cadherin in RA patients [56].

### Collagenases

Matrix metalloproteinase 9 (MMP9), or gelatinase, was >2-fold upregulated in SpA. This enzyme is a known disease activity marker for arthritis and its level increases with a corresponding increase in degradation of extracellular matrix. It has already been identified in RA and SpA cases [57,58]. However, to the best of our knowledge, there are no reports on its relative expression across RA and SpA. Metalloproteinase inhibitor 1 (TIMP1) was found to be >1.5-fold upregulated in RA. Increased levels of TIMP-1 have been observed in synovial fluid samples of RA as compared to psoriatic arthritis and are thus in agreement with our data [59].

### Membrane proteins

SUN domain-containing protein 3 (SUN3), is a nuclear membrane protein with transmembrane and C terminal SUN domains [60]. This protein binds to Klarsicht/ANC-1/Syne homologue domains thereby forming bridges between outer and inner membranes of a nucleus [60]. It was observed to be 3-fold upregulated in SpA and has not been previously associated with any form of arthritis.

### Apolipoproteins

The proteins from serum amyloid protein family identified in this study include serum amyloid P component (APCS; 4-fold higher in SpA) and serum amyloid A (SAA1; 3-fold elevated in SpA). Amyloid deposits in organ systems lead to amyloidosis [61], which is a life-threatening complication in rheumatic diseases and has a prevalence of >5% in rheumatic diseases [61]. These proteins have already been reported in SpAs [62,63].

Additionally, we identified apolipoprotein C-III, C-II and D proteins. A 2-fold upregulation of apolipoprotein D in RA was identified and it was not reported earlier. Apolipoproteins, C-II and C-III types were each found to be 3-fold downregulated in RA and were never identified in SpA. These proteins are critical in cholesterol metabolism and their increased levels in synovial fluid suggest a higher rate of triglyceride and cholesterol transport which increases the risk of atherosclerosis [64].

### Other secretory proteins

Tetranectin (CLEC3B), a secretory glycoprotein of unknown function, was found to be 2-fold upregulated in RA. Its overexpression in serum and synovial fluid of RA as compared to osteoarthritis and SpA have been reported earlier [65]. Vitamin D binding protein (GC), a glycoprotein, was found to be >1.5-fold upregulated in RA and has been reported earlier in synovial fluid and serum samples of RA [66,67]. These proteins are carrier proteins for 25-hydroxyvitamin D3, and are involved in regulation of bone mineral density [68]. Galectin-3 binding protein, (LGALS3BP), an N-linked glycoprotein, was found to be overexpressed 4-fold in SpA (Additional file 3: Figure S1D). This protein has already been identified in RA [69] although not described earlier in the context of SpA. It is considered as a disease activity marker and possibly plays vital role in the activation of synovial fibroblasts [69].

### Validation by ELISA

**Figure 3:**
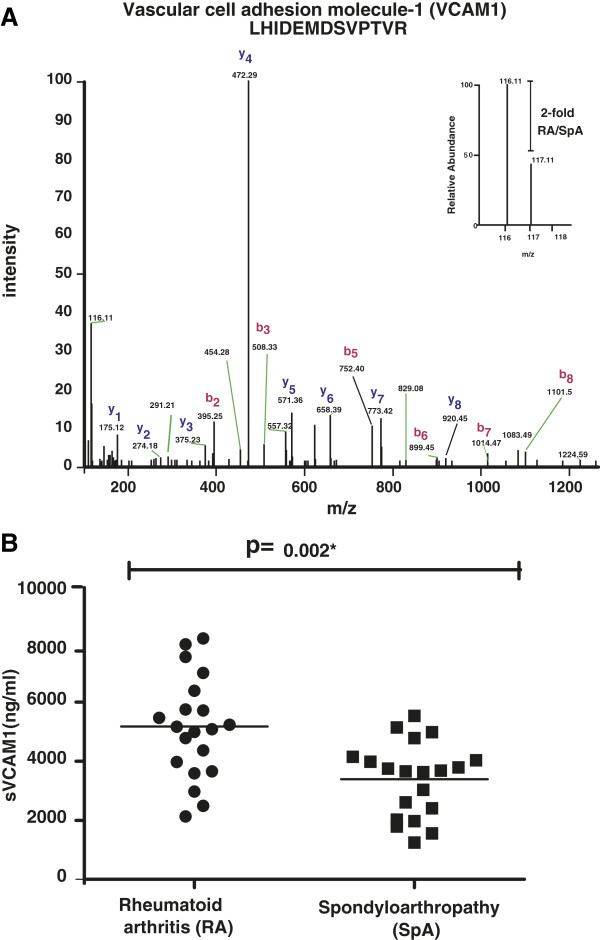
**Upregulation of sVCAM-1 in RA.** MS/MS spectrum of one of the peptides (N-LHIDEMDSVPTVR-C) identified from VCAM-1 with the inset spectrum showing its upregulation in RA **(A)**. Concentration of sVCAM-1 in synovial fluid obtained from RA and SpA patients as measured by ELISA **(B)**.

VCAM-1 has been considered critical for T cell infiltration [70] and is an endothelial dysfunction marker of RA [71]. Elucidation of its dual role in inflammation and endothelial dysfunction in rheumatic disorders has been a major focus for researchers. There are no reports on its differential expression pattern in the synovial fluid in RA and SpA. Given its functional implication in RA and considering the high risk of cardiovascular manifestations, we hypothesized that there should be a significant difference in the expression levels of VCAM-1 across RA and SpA patients. Thus, we carried out sandwich ELISA-based quantification of VCAM-1 in synovial fluid samples of RA (n = 20) and SpA (n = 20) patients. We found that soluble VCAM-1 concentration in RA (2–8.3 μg/ml) was significantly higher than in SpA (1.2-5.7 μg/ml) with a (p-value = 0.002; Mann–Whitney U test). This finding validates our MS-based quantitative data that showed a higher level of VCAM-1 in RA. The MS/MS spectral representation of one of its peptides identified is provided in Figure 3(A) and the relative abundance pattern across RA and SpA from ELISA is shown in Figure 3(B). This data is in agreement with our hypothesis that VCAM-1 may actively participate in the pathogenesis of RA as compared to SpA.

## Conclusions

We implemented a multilectin affinity approach to isolate and quantify glycoproteins from the synovial fluid of patients with RA and SpA. In addition to identifying a number of novel candidates, we found several molecules that were in agreement with previous reports. We validated the overexpression of VCAM-1, a potential inflammatory marker in RA with respect to SpA. In addition to VCAM-1, validation of the overexpression of ECM-1 and LUM in RA and ApoC-II, ApoC-III and complement protein C5 in SpAs could provide newer dimensions to biomarker discovery for rheumatological diseases. A combination of markers such as VCAM-1 and cadherin-5 and LUM and ECM-1 in synovial fluid of RA patients could potentially reflect progression of RA with a corresponding increase in atherosclerosis.

When we compared our data with that from pulldown studies with the lectin jacalin as reported by Saroha et al. [72], we observed that almost all of their data overlapped with the list of upregulated proteins in RA identified by our study. These proteins include protein families such as complement proteins, plasma protease C1 inhibitor, immunoglobulins, alpha glycoproteins and fibrinogen. Future studies could focus on peptide level enrichment, which could provide glycosylation sites in addition to potentially reducing any non-specifically bound proteins.

## Methods

### Collection and processing of synovial fluid samples

Approximately 2–4 ml of synovial fluid was aspirated from knees of 26 patients with RA and 26 patients with SpA and stored in vacutainers containing heparin (BD, NJ, USA) at 4°C. Clinical details of the patients used for are provided in Additional file 4: Table S3. The selection of patients was based on the American College of Rheumatology criteria for RA and on European Spondyloarthropathy Study Group criteria for SpA. The samples were obtained after informed consent from patients and after approval from the ethical committees of the Armed Forces Medical College, Pune, India and Fortis Hospitals, Bangalore, India. The samples were centrifuged at 1,500 g at room temperature for 15 minutes and the supernatants were filtered using 0.22 μm filters (Millipore, Ireland). The filtered samples were stored at −80°C until further analysis. MS-based iTRAQ labeling was performed using the multilectin affinity enriched proteins from 10 pooled synovial fluid samples from each of the two conditions.

### Glycoprotein enrichment

Glycoprotein enrichment was carried out by using a mixture of three agarose bound lectins, Wheat germ agglutinin, Concanavalin A and Jacalin (Vector laboratories, USA), as previously described [32]. Agarose-bound lectins were washed and aliquots from each lectin were combined together to form a suspension. Thereafter, the pooled lectins were split equally and each fraction was mixed with synovial fluid containing 5 mg protein obtained from 10 pooled RA or SpA samples and made up to 5 ml using Tris buffered saline (0.05 M Tris–HCl, pH 7.5, 0.15 M NaCl). After overnight incubation at 4°C, the bound glycoproteins were eluted using competitive elution, performed by a mixture of sugars (100 mM each of M-pyranoside, galactose, melibiose and N-acetyl glucoseamine in Tris buffered saline, pH 7.5). The selection of sugars was based on their specificity towards the three lectins used as per the manufacturer’s instructions. The eluates were then washed and concentrated using 3 kDa MWCO filters (Amicon, Millipore, Ireland). The protein amount was estimated by Lowry’s assay using the Bio-Rad DC method (catalog number 500–0116) and the proteins were stored at −20°C until further use.

### iTRAQ labeling

For each group, 100 μg of enriched glycoproteins were used for iTRAQ labeling. The labeling was carried out essentially as described previously [33]. Denaturation of proteins was carried out by 2% SDS followed by reduction and alkylation with reducing agent and cysteine blocking agents, respectively. Subsequently, the samples were than digested with the modified sequencing grade Trypsin (Promega, Madison, WI, USA) at 37°C overnight. The tryptic peptides from two different sets were labeled using iTRAQ reagents as per manufacturer’s instructions (iTRAQ Reagents Multiplex kit; Applied Biosystems/MDS Sciex, Foster City, CA). We used the 4plex kit for differential labeling; RA and SpA derived tryptic peptides were labeled with 116 and 117, respectively. Labeled peptides were pooled, vacuum-dried and reconstituted in 10 mM KH_2_PO_4_, 20% acetonitrile (pH 2.8) and fractionated by strong cation exchange (SCX) chromatography.

### SCX- based fractionation

SCX chromatography was carried out essentially as described earlier [73]. Briefly, the tryptic peptides were fractionated on a PolySULFOETHYL A column (PolyLC, Columbia, MD, USA) with 200 Å, 5 μm, 200 × 2.1 mm dimensions, using an Agilent’s 1200 HPLC-system (Agilent Technologies Inc., Santa Clara, USA). A linear gradient of increasing solvent B (350 mM KCl in solvent A, pH 2.8) at a flow rate of 200 μl/min with over a period of 70 min was used for fractionation. Peptide fractions were collected using an automatic fraction collector. Complexity of each fraction was determined based on UV absorbance at 214 nm, finally leading to a total of 18 fractions. The fractions were cleaned using custom made C18 stage-tips (3 M Empore high-performance extraction disks) and were subsequently subjected to LC-MS/MS analysis.

### LC-MS/MS analysis

The samples were analyzed on an LTQ-Orbitrap Velos mass spectrometer (Thermo Fisher Scientific Inc., Bremen, Germany) interfaced with Agilent’s 1200 nano-LC system for reverse phase separation of peptides and sample delivery (Agilent Technologies Inc. Santa Clara, USA). Peptides were first enriched on a trap column (75 μm × 2 cm, C18 material 5-10 μm, 100 Å) at a flow rate of 3 μl/min and resolved on a reverse phase analytical column (75 μm × 10 cm, C18 material 5 μm, 120 Å) at a flow rate of 300 nl/min. Peptides were eluted using a linear gradient of 5–30% acetonitrile over 60 min. The electrospray source was fitted with a 5 μm emitter tip (New Objective, Woburn, MA) maintained at 2 kV ion spray voltage. MS data was acquired in a data dependent manner with full scans acquired using the Orbitrap mass analyzer at a mass resolution of 60,000 at 400 m/z and MS/MS scans acquired at a mass resolution of 15,000 at 400 m/z. Twenty most intense precursor ions from a survey scan of each MS cycle, were selected for MS/MS. The fragmentation was carried out using higher-energy collision dissociation (HCD) using 40% normalized collision energy. The fragmented peptides were dynamically excluded for 30 sec. The automatic gain control for full FT-MS was set to 1 million ions and for FT-MS/MS was set to 0.1 million ions with a maximum accumulation time of 200 ms. The lock mass option (m/z, 445.120025) was enabled for accurate mass measurements.

### Data analysis

Proteome Discoverer Beta Version 1.3 (Thermo Fisher Scientific Inc., Bremen, Germany) was used for database searches. A precursor mass range of 350–7000 Da and a signal to noise of 1.5 were used. A combined Mascot (Mascot version 2.2, Matrix Science) and SEQUEST search was done using the Proteome Discoverer suite against the NCBI Human RefSeq database 45 containing 32, 964 entries with known contaminants. Search parameters included trypsin as the enzyme with maximum 1 missed cleavage allowed; oxidation of methionine was set as a dynamic modification while alkylation at cysteine and iTRAQ modification at N-terminus of the peptide and lysine were set as static modifications. Precursor and fragment mass tolerance were set to 20 ppm and 0.1.Da, respectively. Peptide and protein data were fetched using high peptide confidence (1% FDR) and rank one peptide match filters. Reporter ion quantitation node was used for relative expression pattern of proteins based on the relative intensities of reporter ions for the corresponding peptides.

### Enzyme linked immunosorbant assay (ELISA)

We determined the concentrations of sVCAM-1 in synovial fluid of RA and SpA cases using a commercially available ELISA kit (Invitrogen Corporation, Camarillo, CA, USA). Sandwich ELISA was performed with synovial fluid samples from 16 RA and 16 SpA cases, along with 4 additional samples each from the screening phase (thus, n = 20 in RA and SpA). The protocol implemented was as per the instructions given in the kit. The demographic details of the patients have been provided in the Additional file 5: Table S4. The sensitivity of the kit used was <0.5 ng/ml. Statistical analysis was done with the GraphPad Prism version 5.04 (San Diego California, USA). Statistically significant difference among the diseases was calculated by Mann–Whitney U test of the non-normally distributed data. A p-value of 0.05 or lesser was considered significant.

### Bioinformatics analysis

To gain biological insights into the data derived, we carried out a bioinformatics analysis of the protein list. Proteins were classified based on the Gene Ontology (GO)-based molecular class and cellular component features using our in-house resource, Human Reference Protein database, HPRD (http://www.hprd.org/). To determine known glycoproteins in our list, we compared our data with HPRD and the publically available UniProt resource with published literature evidence (http://www.uniprot.org/).

### Data availability

We used the two public data repositories for submitting our mass spec data. Raw files are available online and can be downloaded from Tranche (http://www.proteomecommons.org/tranche/) using the following hash: l + KH4WzubdLKlJnNx2NbDTmCC + Q2SL2SFzXHr4mAfghsZbOzYYBVa + VTOjfxnUg136ByJYXv1JDuZd + Kv8dQIGGbOeQAAAAAAAAIwQ== (URL-http://proteomecommons.org/dataset.jsp?i=77886).

Processed data and the search results including the detailed protein/peptide data can be downloaded from our own resource called the Human Proteinpedia (http://www.humanproteinpedia.org) [74].

## Competing interests

All the authors have read the manuscript and declare to have no competing interests.

## Authors’ contributions

AP, SS, HCH and MB were involved in the conception and study design. MB and LB collected synovial fluid samples. MB, LB, RS, SR, STG and RG carried out the experiments. SMP and NRS were involved in fractionation and mass spectrometry-based data acquisition of the samples. MB, SS and AP wrote the manuscript. MB, SMP, JA and RS were involved in the data analysis and MB carried out data interpretation and statistical analysis. Synovial fluid samples were provided by SS and RJ. RV and MB performed the bioinformatics analysis. BN, TSKP, RJ and HCH critically read and provide input regarding data interpretation and the manuscript. All authors read and approved the final manuscript.

## Supplementary Material

Additional file 1: Table S1 Details of proteins and peptides identified in the study. Click here for file

Additional file 2: Table S2 Details on sub-cellular localization, functional class and whether or not reported as glycoproteins. Click here for file

Additional file 3: Figure S1 Tandem mass spectra of representative peptides identified for Lumican **(A)**, S100-A8 **(B)**, Complement 5 **(C)** and Galectin-3 binding protein **(D)**. Click here for file

Additional file 4: Table S3 Clinical details of patients used in iTRAQ labeling. Click here for file

Additional file 5: Table S4 Demographic details of patients used in ELISA. Click here for file
